# Modulation of circulating osteocalcin-expressing monocytes by glucagon like peptide-1 receptor agonists in type 2 diabetes: an exploratory pilot study

**DOI:** 10.1007/s00592-026-02778-2

**Published:** 2026-09-21

**Authors:** Andrea Carafa, Rosario Luigi Sessa, Rocco Amendolara, Simona Zampetti, Carmen Mignogna, Francesca Sciarra, Luca D’Onofrio, Renata Risi, Mary Anna Venneri, Ilaria Cavallari, Ernesto Maddaloni, Raffaella Buzzetti

**Affiliations:** 1 Department of Experimental Medicine, Sapienza University of Rome, Viale Regina Elena 324, 00161 Rome, Italy; 2 Department of Molecular Medicine, Sapienza University of Rome, Rome, Italy; 3 Diabetes Unit, Department of Internal Medicine, Endocrinology and Metabolism and Infectious Disease, Policlinico Umberto I University Hospital, Rome, Italy; 4 Unit of Cardiology, Department of Medicine, Fondazione Policlinico Universitario Campus Bio-Medico, Rome, Italy

**Keywords:** GLP1-RAs, Metformin, CD14+ OCN+ monocytes, Type 2 Diabetes, Cardiovascular, Exendin-4

## Abstract

**Context:**

The phenotypic drift of circulating monocytes toward osteoblast-like cells expressing osteocalcin (OCN) has been linked to vascular calcification in individuals with type 2 diabetes (T2D). The synergistic action of glucagon-like peptide-1 receptor agonists (GLP-1 RAs) and metformin may reduce the cardiovascular risk by modulating OCN-expressing monocytes, representing a novel mechanism of cardiovascular protection.

**Objective:**

To investigate the impact of GLP-1 RA add-on metformin therapy on the percentage of circulating CD14^+^OCN^+^ monocytes.

**Research design and methods:**

This longitudinal study enrolled patients with T2D receiving metformin monotherapy who either initiated GLP-1 RA therapy (Group A, n = 7) or did not (Group B, n = 28). Clinical parameters and circulating CD14+OCN+ cell levels were assessed at baseline (V0), after 1 week (V1) and after 12 weeks (V2). In vitro experiments were conducted using peripheral blood mononuclear cells (PBMCs) from healthy volunteers (n = 10), treated with metformin or exendin-4 for 24 h.

**Results:**

In group A, the percentage of CD14+OCN+ significantly decreased from baseline (4.5 [2.5–7.6]) to V2 (2.2 [1.73–4.9]% p = 0.019). This reduction was already significant at V1 (p = 0.039). No significant change over time was observed in Group B (p = 0.31). In vitro treatment of PBMCs with metformin significantly reduced the percentage of CD14^+^OCN^+^ cell (− 34.9%, p < 0.0001), whereas exendin-4 yielded a non-significant downward trend (− 8.5%, p = 0.1).

**Conclusion:**

GLP-1 RA add-on therapy to metformin reduces OCN expression in circulating monocytes in patients with T2D. In vitro data suggest that this effect might not result from a direct effect on mononuclear cells, but rather from indirect systemic mechanisms that warrant further investigation. These findings identify OCN-expressing monocytes as a potential novel therapeutic target for GLP-1 RA to reduce cardiovascular risk in T2D.

**Supplementary Information:**

The online version contains supplementary material available at https://doi.org/10.1007/s00592-026-02778-2.

## Introduction

Type 2 diabetes (T2D) is a chronic metabolic disorder that, through a complex interplay of pathological mechanisms, leads to multi-organ damage and significantly increases the risk of cardiovascular disease. Despite significant advancements in therapeutic strategies, cardiovascular events remain the leading cause of death among people with T2D [1, 2]. Therefore, the investigation of the biological mechanisms of cardiovascular disease in diabetes remains a subject of extensive research [3, 4].

Osteocalcin (OCN)-expressing osteoprogenitor cells have been found in higher percentages in patients with cardiovascular disease regardless of diabetes status [5]. Their pro-calcific phenotype suggests a potential contribution to atherosclerosis and vascular calcification. Moreover, recent studies on myeloid calcifying cells (MCCs), a subset of monocytes expressing CD45^+^/CD14^+^ and bone-related markers, highlighted their involvement in arterial calcification and plaque vulnerability in early coronary atherosclerosis [6]. In individuals with T2D, MCCs have been closely associated with cardiovascular disease and ectopic vascular calcification, showing a positive correlation with glycated hemoglobin levels (HbA1c) [7]. Despite therapeutic advancements, the impact of current glucose-lowering drugs on MCC-mediated calcification remains unexplored, representing a critical knowledge gap in cardiovascular risk management [8–13].

Recent pharmacological investigations have explored the broader effects of glucose-lowering agents, including GLP-1 receptor agonists (GLP-1 RAs) and metformin, beyond glycemic control [14–18]. While GLP-1 RAs have protective cardiovascular effects in patients with T2D [19, 20], the molecular mechanisms underlying the positive impact on atherosclerosis remain unclear. Additionally, GLP-1 receptors are expressed in bone tissue, suggesting a possible role in osteoblast differentiation and osteocalcin regulation [15]. Similarly, metformin, a cornerstone therapy in T2D, has demonstrated cardiovascular benefits, in part through AMPK activation, which modulates inflammation and macrophage polarization [19, 20]. Effects of metformin on bone metabolism have also been suggested in preclinical and clinical studies [21, 22].

We hypothesize that GLP-1 RA may reduce the levels of pro-calcific circulating monocytes, as a possible novel mechanism of cardiovascular protection. Therefore, this study primarily aims to assess whether GLP-1 RAs are able to modulate OCN-expressing circulating monocytes in individuals with T2D. Secondary endpoints included in vitro experiments assessing the effects of metformin and a GLP-1 RA on peripheral blood mononuclear cells (PBMCs) obtained from healthy donors.

## Material and methods

### Study design and population

This longitudinal study was conducted at the outpatient clinic of the Diabetes Unit of the Policlinico Umberto I General Hospital, Rome, Italy. Subjects (both males and females) affected by T2D on metformin starting semaglutide, a GLP-1 RA, (Group A) or continuing metformin monotherapy (Group B) were consecutively enrolled from January 2023 to December 2024 if meeting the following inclusion criteria: age > 18 years and < 80 years, on stable monotherapy with metformin (2000 mg/day or max tolerated dose) for at least 6 months before enrollment, HbA1c < 8.0%, body mass index (BMI) < 45 kg/m^2^. Subjects not willing to adhere to study procedures, those with active neoplastic disease, history or active hematologic disease, estimated glomerular filtration rate < 30 mL/min/1.73 m^2^, history or active of hypo- hyperparathyroidism, anorexia, alcoholism, hypogonadism, inflammatory bowel disease, diabetic gastroparesis, previous acute pancreatitis, trauma or chronic immobilization, pregnancy, lactation, ongoing treatment with immunomodulators or corticosteroids were excluded from the study. Enrolled patients were followed up for 3 months and attended three study visits: baseline (V0) and after 7 ± 2 days (V1), and 84 ± 5 (V2) days.

### Clinical data collection

Anthropometric data and clinical information were recorded from medical records for patient stratification, including sex, age, diabetes duration, comorbidities, and biochemistry. BMI was calculated as body weight in kilograms divided by height in squared meters (kg/m^2^). Waist circumference was measured using a non-elastic tape measure, halfway between the lower edge of the lowest rib and the iliac crest on the horizontal plane at the end of normal exhalation and recorded to the nearest 1.0 cm. Blood pressure was measured on the day of clinical examination. The participants were kept resting in a sitting position for at least 5–10 min in a fasted condition, avoiding tobacco use or the intake of coffee or any stimulating beverage before the evaluation. The average of three consecutive measurements was used for the analysis. Hypertension was defined as a systolic blood pressure ≥ 140 mmHg or a diastolic blood pressure ≥ 90 mmHg or any use of antihypertensive drugs. Fasting blood glucose (FBG), glycated hemoglobin (HbA1c), total cholesterol, triglycerides, high-density lipoprotein cholesterol (HDL-c), creatinine were measured with standard laboratory procedures at baseline and at follow-up visits. Low-density lipoprotein cholesterol (LDL-c) were estimated using the Friedewald et al.’s formula [23]. The eGFR was estimated using the CKD-EPI formula, according to Levey et al. [24]. Healthy volunteers without diabetes, and fulfilling the same inclusion/exclusion criteria used for the in vivo study cohort, were also enrolled for the in vitro study at the same healthcare facility (Policlinico Umberto I General Hospital, Rome, Italy). All participants’ data were recorded in an anonymized Excel spreadsheet.

### Peripheral blood mononuclear cells isolation and identification/quantification of circulating OCN^+^ monocytes by flow cytometry

Based on previous reports [25], OCN+ circulating monocytes were identified in the peripheral blood mononuclear cells (PBMCs) isolated in blood samples obtained from fasting blood samples at each visit. PBMCs were isolated using a Ficoll-Paque density gradient, washed twice in 2 ml of PBS+1%FBS, and then incubated for 45 min at 4 °C in the dark with Human Osteocalcin Alexa Fluor® 488-conjugated Antibody (R&D System, Minneapolis, Minesota, United Stated, Cat N IC1419G) and CD14 Monoclonal Antibody (61D3) Super BrightTM 600, eBioscience (Invitrogen Corporation, Waltham, Massachusetts, United Stated, Cat N 63-0149-42) according to manufacturer’s instructions. Samples were then assessed using a CytoFLEX-S flow cytometer (Beckman Coulter, Brea, California, United Stated). Flow Cytometry data were analyzed using CytExpert software (Beckman Coulter, Brea, California, United Stated). Monocytes (MONO) were identified as CD14+ cells, and OCN+ monocytes were identified via the FITC channel. A minimum of 10^5 events were acquired per sample. Gating strategies and data analysis were performed using Cytexpert and FlowJo V10 software.

### In vitro experiments

In vitro studies were conducted on peripheral blood mononuclear cells (PBMC) isolated from blood samples collected from 10 healthy volunteers without diabetes. To evaluate the expression levels of OCN, PBMC were cultured in RPMI-1640 medium (1:1) (Sigma-Aldrich, St.Louis, Missouri, United Stated, Cat. N R8758) according to the manufacturer’s instruction. The medium was supplemented with specific treatments for a 24 h incubation period with 1 mmol/L Metformin (Met) (Sigma Aldrich, Cat N MFCD00012582) and 10 nmol/L Exendin-4 (Exe-4) (Santa-Cruz, Dallas, Texas, United Stated, Cat N SC474611A) and incubated at 37 °C in a humidified atmosphere with 5% CO₂. Untreated cells were included in each experiment. After incubation, cells were harvested, washed, and processed for OCN and CD14 gating by using a CytoFLEX-S flow cytometer (Beckman Coulter, Brea, California, United Stated).

### Statistical analysis

Continuous variables are expressed as median [25th-75th percentile range], while categorical variables are presented as numbers and proportions. Normality was tested graphically (Q-Q plot) and with the Shapiro–Wilk test. The Mann–Whitney test was employed to compare continuous variables between groups, while Chi-squared test and the Fisher exact test were used to compare categorical variables between groups, as appropriate. Friedman test was used to evaluate the longitudinal change of the percentage of CD14^+^OCN^+^ PBMCs over the three time-points of the study. Comparisons between each time point (V1 vs. V0 and V2 vs. V1) were performed with the Wilcoxon test if a significant change was observed over the three time-points.

As this was an exploratory pilot study, no formal sample size/power calculation was a priori performed. However, based on the primary aim of exploring the effect of GLP1-1 RA on the percentage of CD14^+^OCN^+^ circulating cells over 12 weeks, we determined that 7 patients starting a GLP-1 RA (Group A) would provide sufficient power (> 80% at a one-tailed alpha level of 0.05) to detect a large effect size (d ≥ 0.85). To allow a drop-out rate > 20%, we deemed to enrol a total of 10 patients starting a GLP-1 RA. For comparison, we also deemed to enroll at least 30 patients who continued metformin monotherapy (Group B).

All statistical analyses and graphical representations were performed using GraphPad Prism (GraphPad Software LLC, Boston, Massachusetts, USA). *P-*values < 0.05 were considered as statistically significant.

### Ethics

The study was performed in accordance with the Declaration of Helsinki, and the study procedures were approved by the institution ethics committee (Prot. No. 0905/2022). All subjects enrolled in the study signed a written informed consent before being enrolled in the study.

## Results

### Population features

Ten patients starting a GLP-1 RA (group A) and 34 continuing metformin monotherapy (Group B) were enrolled (Supplementary Table 1). Of these, 3 and 6 were lost to follow-up, respectively, leaving 7 participants in Group A and 28 participants in group B with complete data for the final analysis. The clinical features at baseline and after 12-weeks follow-up of the population completing the study are summarized in Table 1**,** stratified by study groups. At baseline, participants starting GLP-1 RA had higher HbA1c (7 [6.7-7.2] vs 6.2 [6.0-6.5] %; 52.9 [51.4-56.8] vs 44.2 [42.0-47.5] mmol/mol, p < 0.001). No differences were observed in age, sex distribution, BMI, lipid profile, blood pressure or eGFR (Table 1). In addition, there were no reported differences between groups regarding diabetes-related complications (Table 1).

**Table 1:** Population features

	V0			V2				
	Group A (n = 7)	Group B (n = 28)	p-value	Group A (n = 7)	Group B (n = 28)	p-value		
			Between groups			Between groups	vs V0 (group A)	vs V0 (group B)
Females, n (%)	2 (22.2)	9 (27.7)	0.86	2 (22.2)	9 (27.7)	0.86	-	-
Age, years	65 [59.5-70.5]	61 [55-72.5]	0.76	61 [55-70]	65 [59.5-71.25]	0.76	-	-
BMI, kg/m^2^	35.2 [27.8-36.0]	29.5 [26.4-34.0]	0,18	34.7 [24.7-36.3]	29.3 [26.5-33.2]	0.36	0.38	0.79
Waist circumference, cm	108.5 [102.0-112.8]	102.0 [94.0-110.0]	0.31	111 [102.0-114.8]	105.0 [96.4-110.6]	0.32	0.31	0.78
Diastolic blood pressure, mmHg	82.0 [76.0-85.3.0]	80.0 [60.0-89.0]	0.96	78.0 [58.0-82.5]	81.0 [71.5-88.0]	0.40	0.34	0.02
Systolic blood pressure, mmHg	140.0 [118.0-156.5]	130 [125.0-140.0]	0.78	142.0 [136.0-152.5]	131.0 [125.0-142.5]	0.14	0.69	0.65
Heart rate, bpm	80.0 [72.0-84.5]	76.0 [72.5-81.5]	0.54	77.0 [73.5-79.0]	80.0 [70.8-85.8]	0.71	0.56	0.46
HbA_1c,_ %	7.0 [6.8-7.4]	6.2 [6.0-6.5]	<0.001	5.9 [5.8-6.0]	6.2 [6.0-6.6]	0.20	0.01	0.92
HbA_1c,_ mmol/mol	52.9 [51.4-56.8]	44.2 [42.0-47.5]	<0.001	52.9 [51.4-56.8]	44.2 [42.0-47.5]	0.20	0.01	0.91
Total cholesterol, mg/dl	164.5 [151.0-180]	166.0 [136.5-191.3]	0.79	142.0 [127-169.5]	170.0 [156.5-187.0]	0.10	0.21	0.55
LDL cholesterol, mg/dl	77.0 [67.5-103.0]	86 [71.8-101.8]	0.97	68.2 [60.0-102.5]	90.0 [65.5-107.5]	0.41	0.47	0.93
HDL cholesterol, mg/dl	36.5.0 [33.5-45.5]	48.5 [41.2-56.5]	0.008	42.0 [37.0-50.0]	50.0 [42.0-59.3]	0.29	0.43	0.20
Triglycerides, mg/dl	135.5 [101.5-301.5]	103.5 [82..5-135.3]	0.11	92.0 [61.5- 130.0]	92.0 [87.8-139.0]	0.71	0.01	0.86
eGFR, ml/min/1,73 m^2^	81.5 [72.5-112.5]	88.5 [73.3-96.5]	0.90	76.5 [74.0-108.0]	82.0[71.3-96.5]	0.85	0.38	0.01
History of CVD, n(%)	1 (14.3)	6 (21.4)	> 0.99	-	-	-	-	-
History of nephropathy, n(%)	0 (0)	0 (0)	> 0.99	-	-	-	-	-
History of retinopathy, n(%)	1 (14.3)	0 (0)	0.2	-	-	-	-	-
History of neuropathy, n(%)	0 (0)	0 (0)	> 0.99	-	-	-	-	-

Baseline (V0) and end-of-study (V2) clinical and biochemical characteristics of study participants treated with GLP-1 receptor agonists (GLP-1 RA)(Group A) or metformin (Group B). Data are reported as median [interquartile range] or number (%), as appropriate. Between-group comparisons at each visit are shown. *p* values indicate statistical significance between treatment groups

After 12-weeks follow-up, participants starting a GLP-1 RA (Group A) showed a significant reduction in HbA1c and Tryglicerides (both p = 0.01 vs baseline) (Table 1). All other clinical and biochemical parameters remained statistically unchanged. In people continuing metformin monotherapy after 12 weeks (Group B) showed a statistically—though not clinically—significant change in diastolic blood pressure, eGFR (p-value for the difference at baseline, respectively: 0.02; 0.01).

### Effect of GLP-1 RA therapy on circulating CD14^+^OCN^+^ PBMCs in individuals with type 2 diabetes

At baseline, the median percentage of circulating CD14⁺OCN PBMCs was 4.5 [2.5-7.6] % in Group A and 8.25 [4.5-18.5] % in Group B (p-value for the difference at baseline: 0.192).

**Fig. 1:**
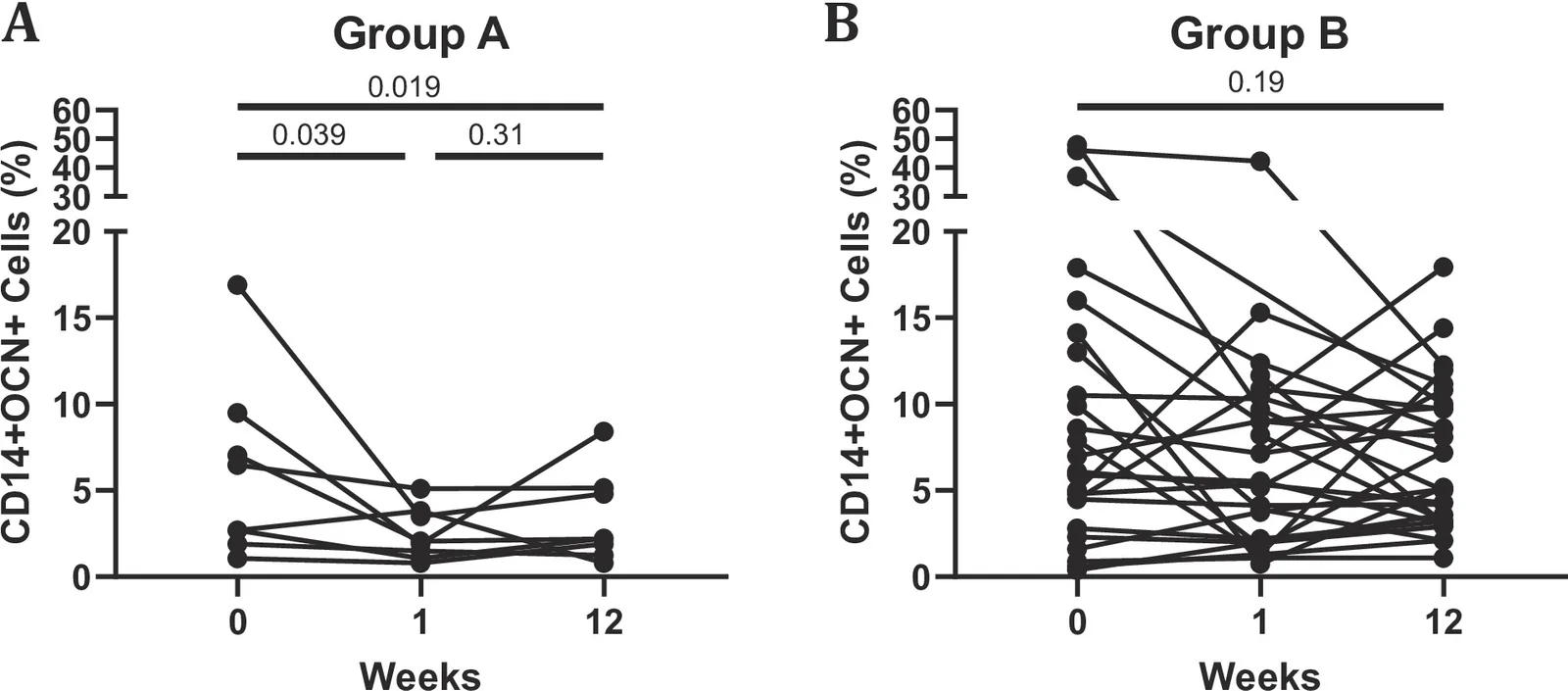
Levels of circulating CD14⁺OCN⁺ PBMCs. Percentage of circulating CD14⁺OCN⁺ PBMCs at baseline and during follow-up in people starting a GLP-1 RA on top of metformin (Group A, Panel **A**) or continuing on metformin only (Group B, Panel **B**). *Friedman test p-value; ^#^Wilcoxon test p-value

Over the 12-weeks follow-up we observed a significant reduction in CD14+OCN+ cell percentage in group A (Friedman p-value: 0.019). Specifically, we observed a significant reduction already at V1 (1.98 [1.3-3.5] % at V1, median Δ_V0-V1_: − 58.2 [27.3–78.9] %, p = 0.0391 vs baseline), which was maintained unchanged at V2 (2.2 [1.73-4.9] % at V2, median change from baseline: − 72.4 [30.8-83.5] %, p = 0.0391 vs baseline; p = 0.31 vs V1) (Fig. 1). The percentage of CD14⁺OCN PBMCs did not significantly change in group B (Friedman p-value: 0.19) (Fig. 1).

### Effects of exendin-4 and metformin on osteocalcin surface expression in human monocytes

**Fig. 2:**
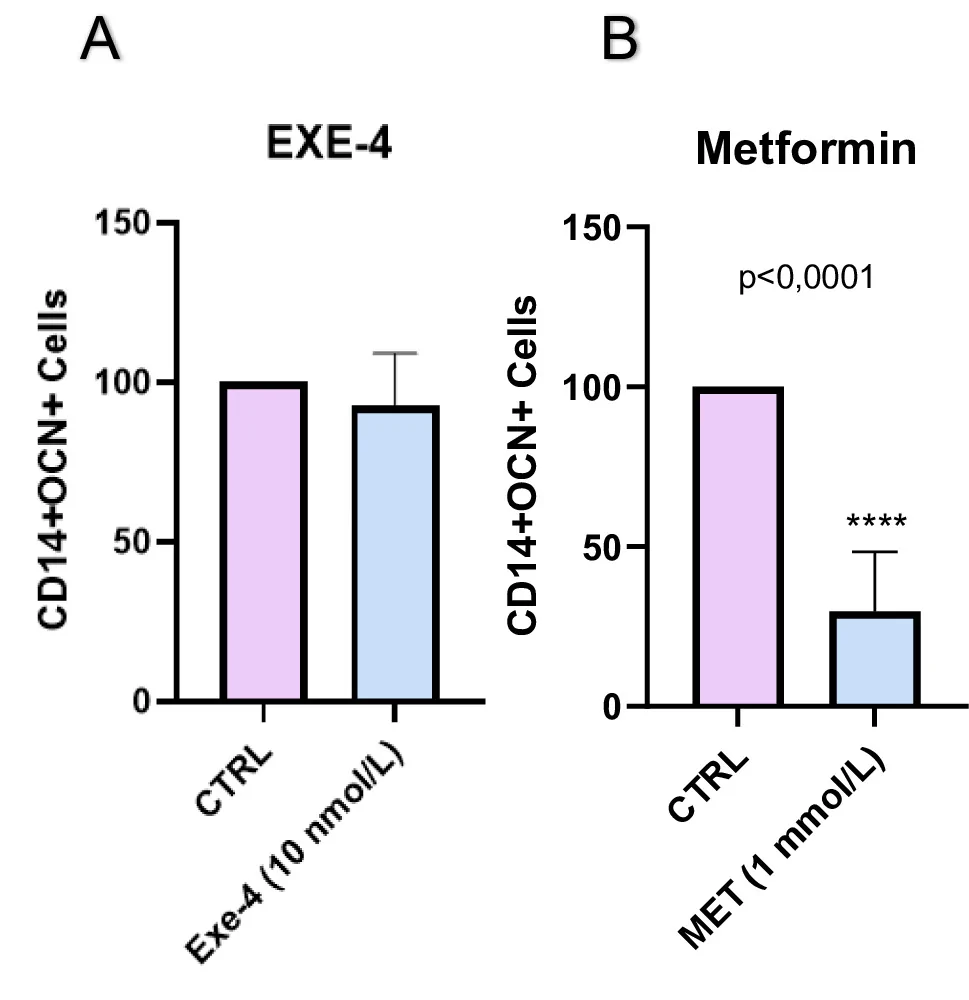
Regulation of OCN Expression by Exendin-4 and Metformin in Cultured PBMCs OCN expression in cultured PBMCs after incubation with Exendin-4 (panel **A**) or metformin (panel **B**)

To evaluate the direct pharmacological modulation of the pro-calcific monocyte phenotype, OCN expression was quantified in PBMCs isolated from apparently healthy volunteers after 24-h exposure to either exenadin-4 or metformin (Fig. 2). Exendin-4 elicited only a modest, non-significant reduction of the median proportion of CD14⁺OCN⁺ monocytes (− 8.5 [− 13.0 to −6.0] %, p = 0.1138) compared to control. In contrast, metformin treatment induced a significant decrease in CD14⁺OCN⁺ cell frequency relative to control (− 34.9 [− 37.0 to −20.1] %, p < 0.0001).

## Discussion

In this pilot study, we explored for the first time the possible action of GLP-1 RA on OCN expression in human monocytes. The clinical data showed an early and significant reduction in CD14^+^OCN^+^ cells in individuals initiating GLP-1 RA therapy in addition to metformin, whereas no significant changes e were observed throughout the study in people continuing metformin monotherapy. However, the in vitro assays failed to demonstrate a direct pharmacological effect of GLP-1 RA in modulating OCN surface expression on monocytes.

Data published so far highlights various pleiotropic effects of GLP-1 RA on different types of immune cells [26–28]. In particular, with regards to cardiovascular disease, one study reported a potential ability of GLP-1 RAs to direct macrophage polarization toward the M2 phenotype, which is typical of alternative activation and therefore has anti-inflammatory and restorative effects. This phenotypic shift could contribute to the antiatherogenic and protective cardiovascular effects of GLP-1 RAs. Another study has demonstrated GLP-1 receptor expression on human monocytes, which decreases as monocytes differentiate into macrophages or foam cells—the latter being characteristic of atherosclerotic lesions [15, 16].

Recent in vitro and in vivo evidence has also shown an action of GLP-1 RA on bone metabolism markers and on osteogenic cells [1], which is in accordance with our findings of an effect of GLP-1RA to modulate OCN expression on circulating monocytes in people with T2D. In this regards, GLP-1 may also affect the fat-bone axis, promoting osteogenic differentiation and inhibiting adipogenic differentiation of bone marrow-derived stem cells (BMSCs), which express the GLP-1 receptor.

OCN is the most abundant non-collagenous protein in the bone matrix and is synthesized by osteoblasts—the same cells involved in atherogenic mechanisms and vascular calcium deposition [29]. Consequently, the pro-calcific phenotypic drift of circulating monocytes, suggested by our pilot data, might be a novel pathway of vascular protection exerted by GLP-1 RAs [15, 16]. Importantly, findings obtained in the clinical study were apparently not confirmed in the in vitro study. These contrasting results might suggest that GLP-1 RA might modulate OCN expression indirectly. In this regards, GLP-1 RA have been shown to acutely improve metabolic control and decrease systemic inflammation [30], which might impact on the procalcific drift of monocytes and on OCN levels in type 2 diabetes [31, 32], although our study was not designed nor adequately powered to investigate for mediators of the GLP-1 RA effects.

Paracrine signaling might also be relevant to explain our findings: GLP-1RAs promote osteogenic differentiation and OCN expression in mesenchymal stem cells and osteoblasts via the Wnt/β-catenin pathway, which may influence monocyte phenotype and function in vivo through local bone marrow microenvironment interactions [33, 34]. Immune modulation is a key mechanism, as GLP-1RAs suppress inflammatory signaling in monocytes and other immune cells, which can relieve inflammation-mediated suppression of OCN expression [35]. These indirect effects might be responsible for the discrepancies between clinical and in vitro findings, as in vitro studies lack the complex systemic and paracrine interactions present in vivo, including the influence of metabolic status, circulating cytokines, and cross-talk between bone, adipose, and immune tissues [36, 37].

On the other side, the in vitro data on metformin indicate that this evergreen anti-diabetes molecule might decrease the pro-calcific phenotypic drift of monocytes through a direct effect. Since all study participants were already on metformin for > 6 months before enrollment, we were not able to evaluate the impact of metformin therapy in the clinical study. Indeed, it is plausible hypothesizing that the efficacy of metformin reaches a plateau over time, preventing any further observable reduction. Indeed, this study was not designed to explore the effect of metformin on the monocytes’ surface OCN expression in patients with type 2 diabetes. Nonetheless the finding of the in vitro experiments paves the path for future clinical investigations exploring this novel potential mechanism of action of metformin to reduce cardiovascular morbidity in diabetes [38, 39].

The study has some limitations. First, the number of patients starting a GLP-1 RA therapy was small. Second, the observational design, which precluded randomization, the use of a placebo and the enrollment of drug-naïve patients. Larger interventional studies are therefore needed to confirm our findings. We also acknowledge that we cannot exclude that the discrepancy in the results obtained in the clinical study and in the in vitro experiments might be caused by the different GLP-1 RA used. Indeed, the GLP-1 RA used in the in vitro experiments (exendin-4) differs in structure and in some pharmacodynamic properties from semaglutide, GLP-1 RA used by patients enrolled in the clinical study.

Nonetheless, given the exploratory nature of this pilot study, it may serve as the basis for expanded research into an important cardiovascular risk factor using drugs that are already well-established in the management of T2D.

## Conclusion

In conclusion, this pilot study provides the first evidence that GLP-1 RA reduce osteocalcin-expressing circulating monocytes in people with type 2 diabetes, suggesting a novel mechanism through which these agents may confer cardiovascular protection by modulating the pro-calcific phenotypic drift of myeloid cells. While metformin appears to exert a direct effect on monocyte osteocalcin expression in vitro, the mechanisms underlying GLP-1 RA action may be more complex and indirect, involving systemic metabolic improvements and immune modulation. Given that myeloid calcifying cells are associated with coronary calcification and plaque vulnerability in early atherosclerosis, and that no targeted therapies currently exist for this pathological process, these findings warrant further investigation in larger, randomized controlled trials to establish whether modulation of OCN-expressing monocytes represents a viable therapeutic target to reduce cardiovascular morbidity and mortality in diabetes.

## Supplementary Information

Below is the link to the electronic supplementary material.

Supplementary Material 1
