# Supplementary material for: Modulation of circulating osteocalcin-expressing monocytes by glucagon like peptide-1 receptor agonists in type 2 diabetes: an exploratory pilot study
Source: Acta Diabetol. 2026 Sep 21;63(12):2189–96. doi: 10.1007/s00592-026-02778-2 (PMC13769745; doi:10.1007/s00592-026-02778-2)
Supplement: Supplementary file 1 [file 592_2026_2778_MOESM1_ESM.docx]

**Supplementary table. *Population features of the whole enrolled population, including participants lost to follow-up***

Baseline (V0) clinical and biochemical characteristics of study participants treated with GLP-1 receptor agonists (GLP-1 RA)(Group A) or metformin (Group B). Data are reported as median [interquartile range] or number (%), as appropriate. Between-group comparisons at each visit are shown. p values indicate statistical significance between treatment groups.

|  | **V0** | | |
| --- | --- | --- | --- |
|  | **Group A**  n=10 | **Group B**  n=34 | **p-value** |
|  |  |  | **Between groups** |
| Females, n (%) | 3 (30) | 10 (29.4) | 0.65 |
| Age, years | 71.5 [65.5-75.0] | 65.0 [57.0-82.5] | 0.67 |
| BMI, kg/m^2^ | 33.2 [27.8-36.4] | 29.0 [26.6-32.9] | 0.16 |
| Waist circumference, cm | 108 [103.0-114.0] | 102.0 [94.0-109.0] | 0.10 |
| Diastolic blood pressure, mmHg | 84.0 [74.0-91.5] | 80.0 [75.0-84.5] | 0.46 |
| Systolic blood pressure, mmHg | 138.0 [131.3-143.0] | 120.0 [85.5-130.0] | 0.001 |
| Heart rate, bpm | 79.0 [67.3.0-85.8] | 74.5 [71.3-80.8] | 0.61 |
| HbA_1c,_ % | 6.9 [6.7-7.0] | 6.2 [6.0-6.5] | p<0.0001 |
| HbA_1c,_ mmol/mol | 52.4 [50.4-52.9] | 44.2 [42.0-47.5] | p<0.0001 |
| Total cholesterol, mg/dl | 157 [133.0-171.3] | 166.0 [137-191.5] | 0.28 |
| LDL cholesterol, mg/dl | 77.0 [59.5-94.0] | 91.5 [72.8-103.3] | 0.31 |
| HDL cholesterol, mg/dl | 39.0 [34.8-43.3] | 48.0 [41.5-55.5] | 0.003 |
| Triglycerides, mg/dl | 100.0 [82.0-135.5] | 88.0 [73.5-94.5] | 0.13 |
| eGFR, ml/min/1,73 m^2^ | 81.5 [67.0-88.3] | 88.0 [73.5-94.5] | 0.43 |
| History of CVD, n(%) | 1 (11.1) | 7(26.9) | 0.66 |
| History of nephropathy, n(%) | 1 (11.1) | 0 (0) | >0.99 |
| History of retinopathy, n(%) | 1 (11.1) | 0 (0) | 0.23 |
| History of neuropathy, n(%) | 0 (0) | 0 (0) | 0.23 |
